# Glioblastoma Multiforme in a Patient With Alpha-1-Antitrypsin Deficiency

**DOI:** 10.7759/cureus.47371

**Published:** 2023-10-20

**Authors:** Curtis Ober, Rojin Esmail, Damian Casadesus

**Affiliations:** 1 Internal Medicine, Jackson Memorial Hospital, Miami, USA; 2 Internal Medicine, Ross University School of Medicine, St. Michael, BRB

**Keywords:** cranial neurosurgery, temozolomide, metabolic encephalopathy, alpha-1-antitrypsin deficiency, glioblastoma idh-wildtype

## Abstract

Alpha-1 antitrypsin (A1AT) is a common genetic disease caused by a mutation in the SERPINA1 gene, predisposing patients to severe premature lung and liver disease. Higher expression of SERPINA1 has been associated with a poor prognosis in patients with high-grade glioblastoma. We present a woman in her 70s with a history of A1AT deficiency treated with weekly plasma-purified A1AT infusions, who presented with metabolic encephalopathy. A CT scan of the brain obtained during admission revealed a left frontal lobe mass measuring 1.1 cm. A craniotomy and resection of the lesion were performed, and the pathology studies revealed a glioblastoma multiforme, WHO grade IV. She is currently healing and awaiting treatment with temozolomide with concomitant radiation and tolerating treatment well.

## Introduction

Alpha-1-antitrypsin is an acute-phase protein encoded by the SERPINA1 gene on chromosome 14, whose primary function is to protect tissue from enzymes released during inflammation. Pathogenic variants in SERPINA1 are known to cause A1AT deficiency (A1ATD), leading to lower protease inhibitor activity in serum [1]. In adults, A1ATD has been associated with the early onset of obstructive pulmonary disease, liver cirrhosis, and hepatocellular carcinoma. Some studies have suggested an increased risk of non-hepatic cancer types in patients with A1ATD [2-5]. Research has established that human alpha-1 antiproteinase confers a decreased rate of decline in FEV1 in patients with moderate airway restriction and may offer a survival benefit in patients with severe airway restriction [6]. In a longitudinal study by Hiller et al., the incidence of cancer, including brain cancer, was higher in patients with A1ATD compared to the general population [7].

Glioblastoma multiforme (GBM) is the most prevalent brain cancer in the adult population, with an exceedingly poor prognosis. SERPINA1 has been demonstrated in glioma and GBM cell lines, and higher expression in grade IV high-grade glioma indicates a poor prognosis [8]. We present a patient with a history of A1ATD and high-grade GBM.

## Case presentation

A woman in her 70s with a medical history of A1ATD, currently undergoing weekly infusions of human pooled alpha-1 antiproteinase, presented to the emergency department with altered mental status. Her son described that she began with uncontrollable shaking and seizure-like activity but became unresponsive and was transferred to our institution.

Upon arrival at the emergency room, the patient was lethargic. Vital signs showed a heart rate of 129/minute and a blood pressure of 169/119 mmHg. During the physical examination, the patient appeared ill. The cardiopulmonary exam revealed diminished breath sounds bilaterally, while abdominal examinations were normal. The patient was oriented to person and time and followed commands. Initially, the patient exhibited right upper extremity flexion with the index finger pointing up, right head deviation, and right gaze, followed by secondary generalization to tonic-clonic seizure. This self-resolved in 1-2 minutes, with a post-ictal state lasting 5 minutes.

**Figure 1:**
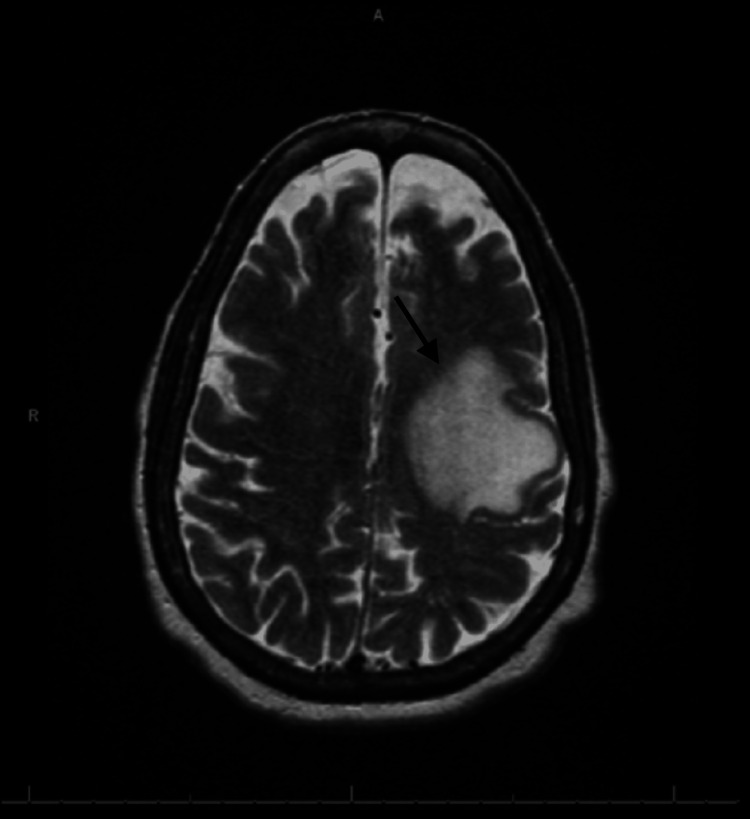
Magnetic resonance imaging of the brain with contrast revealed a lesion in the left frontal lobe with surrounding vasogenic edema and mass effect.

**Figure 2:**
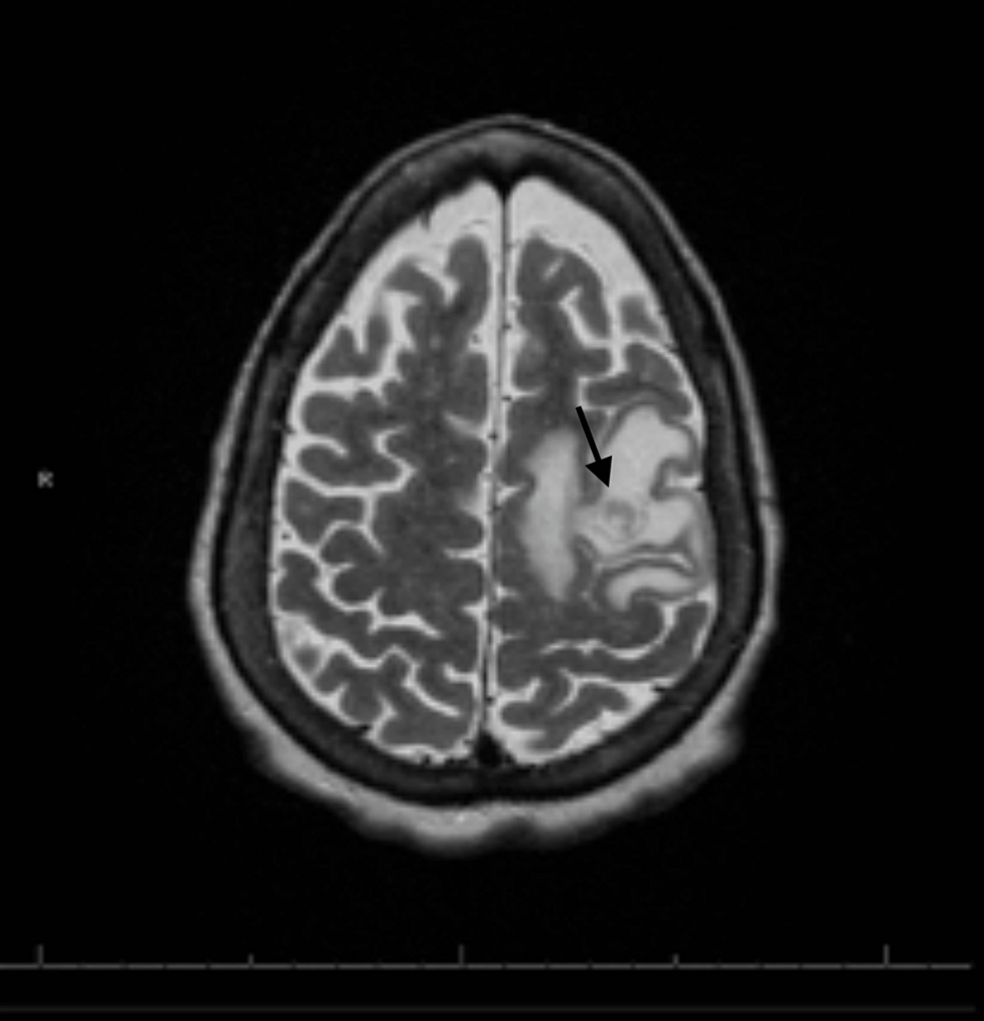
Magnetic resonance imaging of the brain on T2/FLAIR (fluid attenuated inversion recovery) sequence demonstrating central necrotic core characteristically observed in glioblastoma multiforme.

Laboratory studies were abnormal, with lactic acid measuring 6.3 mmol/L and leukocytosis with a predominance of neutrophils. A CT scan of the brain revealed a 1.1 cm heterogeneous, slightly hyperdense intra-axial lesion in the high left frontal lobe, accompanied by surrounding vasogenic edema and mass effect. A CT angiography of the chest was performed, revealing mucous plugs consistent with her history of bronchiectasis, as well as bilateral small hilar lymph nodes that raised concerns for metastatic disease. MRI of the brain, aimed at providing a better characterization of the cranial lesion, showed a brain mass measuring 2.0 x 1.7 x 2.7 cm (Figure 1). MRI of the brain with contrast also demonstrated a heterogeneously enhancing mass with a central necrotic core (Figure 2).

**Figure 3:**
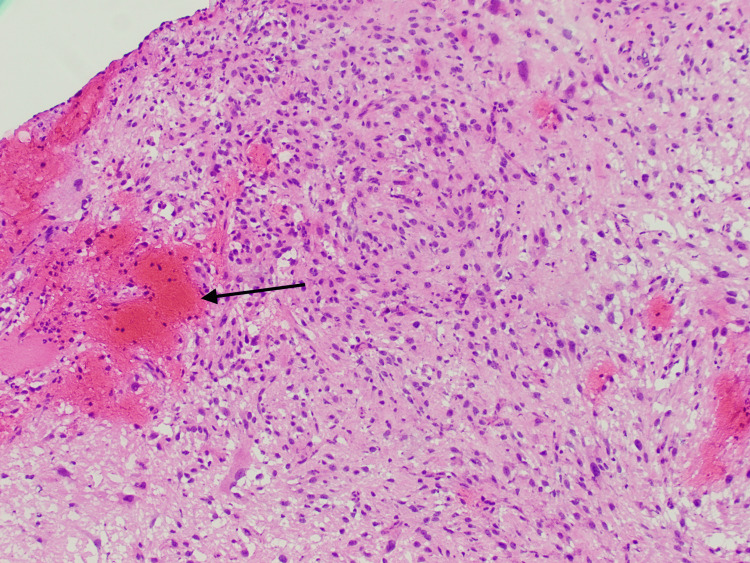
Histological evaluation of glioblastoma multiforme. Black Arrow: a hyperemic blood vessel as a component of glioblastoma multiforme.

**Figure 4:**
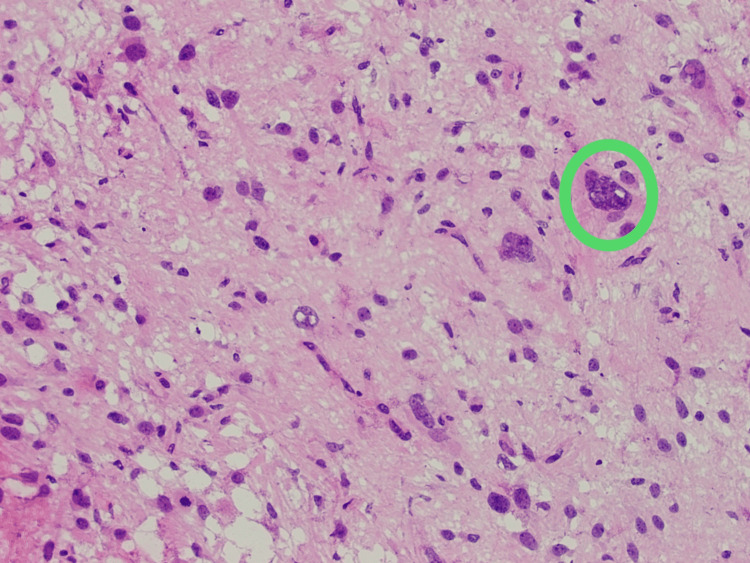
Histological evaluation of glioblastoma multiforme. Green circle: demonstrates multinucleated cells with marked nuclear atypia and numerous mitotic figures.

There was a debate about neurosurgical gross total resection, CT-guided core needle biopsy of the hilar lymph nodes, or mediastinoscopy with lymph node biopsy. Initially, due to the patient's mediastinal lymph nodes, the metastatic brain lesion was thought to be a secondary metastatic site; therefore, tissue sampling of the primary tumor was proposed. After consultation with cardiovascular surgery, she was deemed to be at high risk for surgical complications with mediastinal sampling. CT-guided biopsy of the lymph nodes was also considered to be high risk due to her significant bronchiectasis and the risk for pneumothorax, as well as the consideration of the small size of the target lymph node, measuring only 8 mm. Ultimately, the decision was made to perform a gross total resection of the mass by the neurosurgery team. Pathology studies of the biopsy were consistent with GBM WHO grade IV, with both isocitrate dehydrogenase 1 and 2 testing negative. Methylguanine-DNA methyltransferase (MGMT) was found to be unmethylated. Histological evaluation revealed multinucleated cells with marked nuclear atypia and abundant mitotic figures, consistent with high-grade glioma, as well as a hyperemic blood vessel as a component of the tumor (Figures 3, 4).

The Karnofsky Performance Scale (KPS) has been widely used to assess patient functional status and determine the potential benefits of surgery, radiation, and chemotherapy in patients with GBM [9]. Our patient's functional capability placed her on the KPS scale at 60, as she was able to live independently and care for herself but did require occasional assistance from her children. Her Modified Rankin Scale score was 0, indicating that she did not suffer any residual neurological or motor deficits after her initial seizure-like activity. Initially, she was treated with a dexamethasone taper as well as prophylactic levetiracetam. She has since discontinued the use of dexamethasone.

Due to the patient's age and comorbidities, we discussed various treatment options, including systemic oral chemotherapy, radiotherapy, and palliative care, with the patient and her family. Our patient chose to proceed with concomitant radiotherapy and systemic chemotherapy. Currently, she is receiving treatment with radiotherapy and temozolomide and will subsequently be treated with temozolomide as a single agent.

## Discussion

A1ATD is a genetic disease with a high prevalence and a wide distribution of phenotypic expression. Well-documented sequelae of A1ATD include bronchiectasis and liver cirrhosis. The only well-established cancer indirectly linked to A1ATD is hepatocellular carcinoma. A1ATD may potentially increase the risk of other cancers such as lung, colorectal, gastric, and bladder cancer, leading to a poor prognosis or being associated with a negative impact on invasiveness and metastatic capacity [2-6].

In a study of the Azeri population in Iran, it was indicated that squamous cell carcinoma of the esophagus exhibited heightened levels of alpha-1-antitrypsin expression within the tumor. It is possible to propose that in individuals with A1ATD, the excessive production of this already defective protein might contribute to an escalated susceptibility to cancer [10].

Only one study describes the relationship between A1ATD and central nervous system cancer. Hiller and coworkers reported the presence of non-liver cancers in 14 patients with A1ATD, with only one of the patients having central nervous system cancer involving the meninges (personal communication). To our knowledge, this is the first reported case of a patient with GBM and A1ATD in the literature. In countries with a high incidence of A1ATD, further studies should be conducted to identify the association between these two medical conditions.

## Conclusions

We have presented a case of a patient with a history of A1ATD and high-grade GBM, which raises intriguing questions about a potential association between these two conditions. While further research is needed to establish a definitive link, the presence of A1ATD in this patient with GBM suggests the possibility of a relationship between them. Exploring the underlying mechanisms and conducting additional studies in regions with a high prevalence of A1ATD may provide valuable insights into the association between A1ATD and central nervous system cancers, such as GBM.
